# A Framework to Assess Food Insecurity Responses Among Colleges and Universities

**DOI:** 10.3390/nu18081169

**Published:** 2026-04-08

**Authors:** Sara R. Gonzalez, Kate Thornton, Alicia Powers

**Affiliations:** 1 Department of Nutritional Sciences, College of Human Sciences, Auburn University, Auburn, AL 36849, USA; 2 Department of Human Development and Family Science, College of Human Sciences, Auburn University, Auburn, AL 36849, USA; 3 Hunger Solutions Institute, College of Human Sciences, Auburn University, Auburn, AL 36849, USA; arp0042@auburn.edu

**Keywords:** food insecurity, college students, organizational behavior, framework development

## Abstract

**Background/Objectives:** Food insecurity affects college students at nearly twice the rate of US households, with documented impacts on student academic performance, physical and mental health, and socialization. While frameworks exist to conceptualize general food insecurity and food insecurity in specific contexts, researchers and practitioners lack resources to guide system-level responses to food insecurity on college and university campuses and assess those responses. In this study, we aimed to develop and validate a simple yet comprehensive framework for assessing food insecurity responses within the context of higher education. **Methods:** We adapted an eight-phase process for framework development: (1) map selected data sources within the multidisciplinary literature, (2) read and categorize selected sources, (3) identify and name concepts, (4) deconstruct and categorize concepts based on their features, (5) group similar concepts together, (6) synthesize concepts into a framework, (7) validate the framework using expert panel review, and (8) revise as necessary. **Results:** The developed Campus Food Aid Self-assessment (CFAS) framework consists of six dimensions: Student Services and Supports; Involvement; Advocacy; Awareness and Culture Efforts; Education and Training; and Research, Scholarship, and Creative Works. Expert panelists (*n* = 7) reviewed the proposed framework and confirmed the clarity, comprehensiveness, and representativeness of the proposed dimensions, conceptual definitions, and operational variables. **Conclusions:** With a comprehensive yet accessible structure, the CFAS framework supports the development, coordination, and improvement of campus-based strategies to address food insecurity and support positive student outcomes.

## 1. Introduction

Food insecurity describes the “limited or uncertain availability of nutritionally adequate and safe foods, or limited or uncertain ability to acquire acceptable foods in socially acceptable ways” [1]. During the past decade, more than one in 10 households experienced food insecurity in the United States (US) [2]. Recently, studies have drawn attention to the prevalence of food insecurity among college and university students, with most studies reporting food insecurity prevalence among college students at more than twice the rate of US households [3,4]. Students most likely to experience food insecurity include those who are non-White [5,6,7], first generation college students [6,7,8], working [5,8], low income/Pell grant [3,6,7], living off campus [6,8], or receiving Supplemental Nutrition Assistance Program (SNAP) benefits [3,8]. Researchers have identified negative effects of food insecurity on college student educational achievement [7,8], socialization [9,10], physical health [8,10], and mental health [5,11].

Stakeholders have implemented a variety of responses to student food insecurity. SNAP is the largest federal nutrition assistance program, providing funds to eligible households to purchase staple groceries [12]. Because SNAP requires able-bodied participants with no dependents to work at least 20 h per week, most full-time college students are not eligible for the program [13]. Additionally, a recent report found only 43 percent of eligible students participated in the program [3].

With the first article about campus food insecurity being published in 2009 [14], most college campus responses to student food insecurity have developed during the past decade. While national data on campus responses to food insecurity is limited, campus food pantries have become one of the most common interventions. The College and University Food Bank Association (CUFBA) was founded in 2012 with 15 campus food pantry members and grew to more than 800 campus food pantries by 2021 [15,16]. Additionally, a study of more than 100 two- and four-year colleges and universities found 88 percent of responding campuses had a food pantry [17]. Another study of 108 college campus representatives found 103 had some sort of campus food security program, and of those respondents, 97.1 percent had a campus food pantry [18].

While food pantries and direct provisions are most common, campus responses extend beyond food pantries. In the same study that found 97.1 percent of campuses with food security supports had a campus food pantry, 47.6 percent of campuses had a meal swipe sharing or scholarship program, 43.7 percent had campus community gardens, and 14.6 percent utilized a mobile food sharing application. Additionally, 29.1 percent reported ‘other’ responses, including SNAP outreach, nutrition education, cooking classes, free community meals, sponsored meal plans, and food recovery efforts [18].

These findings were mirrored in the landmark Government Accountability Office report on food security, with officials identifying education of the campus community (including awareness building, faculty and staff training, nutrition education, and financial literacy education), direct food provision, emergency financial aid, centralization and coordination of student services, and research/evaluation as key campus responses to food insecurity [3]. A recent systematic review on the evaluation of campus-based food insecurity programs also identified multiple categories of assistance, with direct food and financial provision being the most common, followed by support services and resources (education and health professional services) [19].

### Need for a Framework to Assess Campus Food Insecurity Response

The complex risk factors, experiences, and impacts associated with food insecurity, particularly among college students, require a multifaceted and systematic response [6,20,21]. While food pantries are by far the most common response to campus food insecurity [18], they fall short of the comprehensive action needed to address the needs of food insecure students. Furthermore, studies of food insecure students have revealed student suggestions that move beyond the food pantry to nutrition and budgeting classes, centralization of services, and increased awareness of resources [22,23,24]. However, practitioners (student support providers, student affairs staff, etc.) lack evidence-based resources to guide systematic responses to student needs that leverage all resources available within college campus environments. While the socio-ecological model describes determinants of health and behavior and is often used to describe barriers and predictors to college student food insecurity [25,26], these typically describe impacts of systems on the individual rather than the behavior of systems themselves. To our knowledge, no framework exists to support understanding or assessment of systematic responses to food insecurity by college campuses through the lens of higher education systems or organizational behavior theory.

Given the complexities of food insecurity, higher education, and food insecurity within higher education, we aimed to develop and validate a simple yet comprehensive framework for assessing food insecurity responses within the context of higher education. The framework was intended to promote understanding and prioritization of campus food insecurity responses, including resources, practices, and policies. IRB approval was received by the authors’ institution before beginning outreach or data collection for framework validation.

## 2. Overview of Relevant Frameworks and Models

### 2.1. Socio-Ecological Model

Ecological systems theory describes how a person’s development and behavior are shaped by interactions with varying levels of their environment [27] and has been applied as the Socio-Ecological Model (SEM) for efforts associated with health-related behavior and promotion [28]. The SEM has been used frequently to study student behavior, experiences, and outcomes on college campuses and to design interventions addressing student wellbeing [29], making it a highly relevant model for examination of intervention and response. The model has relevance to the many interconnected factors affecting student basic needs and has been used to analyze barriers to food resources [26], predictors of food insecurity [25], and specific responses to food insecurity [20]. A systematic review of college campus efforts to address student food insecurity connected campus interventions with corresponding socio-ecological constructs ranging from intrapersonal/individual interventions (e.g., food scholarships and meal swipe programs) to institutional (campus gardens) and policy (comprehensive, campus-wide student support initiatives) level interventions [30].

The Center for Equitable Higher Education (CEHE) developed the ReImagine the Student Experience (RISE) framework based on the SEM to describe the contextual factors affecting student basic needs challenges and strategies to address them. The RISE framework features five nested levels that correspond with the levels of the SEM: (1) individual student, (2) interpersonal, (3) higher education settings, (4) community settings, and (5) societal. The individual student level describes the specific needs and challenges affecting each student, such as food security or mental health challenges. The interpersonal level describes the interactions between students, individuals in the higher education system, and others outside of the school environment, which can drive experiences of stigma. The higher education settings level addresses the impact of institutional policies and practices ranging from basic needs task forces to faculty training and ongoing assessment of programs. At the community settings level, students may engage with or struggle to access resources beyond the campus, whether that be housing, work, or other community resources. Finally, the societal level addresses federal and state policy practices that influence student basic needs [31].

### 2.2. College Food Pantry Concept Map

Few studies have attempted to map the approach to student food assistance through the lens of campus providers. Reppond et al. used a concept mapping approach to answer the question “How does your campus pantry best serve students and foster their success?” [32]. Six thematic clusters based on responses from 28 food pantry representatives included accessibility, available items, student success, support, partnerships, and awareness. While food pantries are far from the only campus response to student food insecurity, they are the most common [17], and the concept map provides critical insight into perspectives of campus representatives leading the most frequently implemented response to student food insecurity at the time this framework was developed.

The accessibility cluster focused on processes and standards influencing student use and accessibility of the food pantry, such as location, intake processes, hours of operation, allowed number of visits, and efforts to create a welcoming, stigma-free environment. The available items cluster described the range of offerings from food pantries and priorities for those offerings, such as healthfulness, diet diversity, or the availability of items and services beyond food. The student success cluster took a holistic perspective to the physical and psychological needs of students to succeed in the classroom and beyond, including academic success, mental health, and basic needs. The fourth concept cluster, support, focused on information sharing, referrals, and connection with additional resources such as education, community-based resources, and SNAP. Beyond campus, the partnerships cluster described on- and off-campus partnerships to improve sustainability and diversity of resources offered. Finally, the sixth cluster, awareness, described dissemination efforts to increase awareness of food insecurity and resources associated with food insecurity while reducing stigma [32]. These clusters help to inform priorities in the development process for campus food insecurity supports, particularly food pantries, and demonstrate the complexity of overlapping or even competing priorities in the development of campus responses.

### 2.3. Organizational Behavior in Higher Education

To examine and understand campus responses to food insecurity, it is important to consider organizational behavior. Berger and Milem’s integrated model for understanding organizational behavior in higher education provides insight into ways organizational behavior shapes a campus environment [33]. It draws on and expands upon Birnbaum’s four models of higher education functioning [34]. Organizational behavior in higher education is defined as “the actions of organizational agents (faculty, administrators, and staff) at a college or university” to serve collective organizational interests, as organizations do not act on their own [35]. Because responses to college student food insecurity are made up of the collective actions of individuals typically serving interests of their college or university (e.g., student retention, success, wellbeing, etc.), organizational behavior theory provides a useful foundation for examining these collective actions. Berger and Milem’s model consists of five dimensions of organizational behavior: bureaucratic, collegial, political, symbolic, and systemic [33,35].

The bureaucratic dimension emphasizes the formal structure of a college or university, including rules, goals, and hierarchy [33]. This includes campus policies relating to food insecurity and institutional structures that exist to support students in need. The collegial dimension focuses on collaboration and participation in the development of goals and decisions [33]. From the perspective of campus food insecurity, this includes the involvement and collaboration of various stakeholders on campus, including faculty, staff, administrators, and students. The political dimension describes the promotion of various interests and competition for limited resources [33]. Because colleges and universities have limited resources, assets necessary for supporting food insecure students (money, human capital, time, space, etc.) often compete against other campus interests. Advocates for food insecure students can play an important role in securing resources necessary for campus response. The symbolic dimension of organizational behavior describes the development of meaning in an organization through stories, symbols, or tradition [33]. Qualitative studies describe how food insecure individuals have expressed frustration, embarrassment, and feelings of low self-worth due to weak or non-existent food security supports from institutions, indicating a campus’s response to food insecurity may have a symbolic meaning to students [9,36,37]. Finally, the systemic dimension focuses on the importance of organizational systems and their interactions with external systems in shaping the environment, norms, and expectations for a campus [33]. College and university responses to food insecurity take place within complex systems. An assessment of responses to student food insecurity must consider the full campus environment and consider student experiences within existing campus systems.

## 3. Materials and Methods

### 3.1. Framework Development

A conceptual framework provides a set of interconnected concepts that collectively offer comprehensive understanding of a phenomenon [38]. We adapted the eight-phase process developed by Jabareen [38], which uses the foundations of grounded theory [39] for framework development: (1) map selected data sources within the multidisciplinary literature, (2) read and categorize selected sources, (3) identify and name concepts, (4) deconstruct and categorize concepts based on their features, (5) group similar concepts together, (6) synthesize concepts into a framework, (7) validate the framework, and (8) revise as necessary.

For phase one, we reviewed the literature on campus food insecurity, food insecurity response, key tenets of higher education, and relevant theories. Because campus food insecurity response has grown rapidly, with a variety of organizations and instutituions working to address this issue, both peer-reviewed and grey literature were included in the review. PubMED, EBSCOhost, and Google Scholar were used to identify peer-reviewed sources. Search terms for review included food secur*, food insecur*, and hunger WITH/AND college, university, campus, higher education, college student, response, or campus response. Additionally, organizational behavior was searched WITH/AND college, university, or higher education. Grey literature included governmental and non-governmental reports, online databases, theses and dissertations, and conference presentations. Google search was used to identify grey literature using the search terms described above. Campus food insecurity sources were included if they addressed food insecurity response by a higher education institution and the food insecurity response was directed toward students. Studies that only addressed food insecurity prevalence or did not include college student populations were excluded.

In phase two, sources were categorized using hybrid thematic coding processes. An initial code list was developed a priori based on the purpose of the study, known efforts in food insecurity response, and organizational behavior theory. Given the intent of this framework to support understanding and assessment of institutional level actions to address college student food insecurity, organizational behavior theory was selected as a foundation for coding and categorization. Coding prioritized observable campus actions with distinct application to food insecurity response. An iterative process was used to review coding and categorization decisions in phase two. Any discrepancies in coding were resolved using a consensus process with a focus on alignment with foundational theories and applicability across multiple campus contexts. This process was repeated until agreement was reached and emergent themes were identified.

In phases three through five, the framework dimensions were developed from the categories identified in phase two to provide a clear and comprehensive structure for assessing food insecurity responses within the bounds and systems of higher education. Conceptual definitions were then developed to define the content domain simply and clearly, and operational variables were identified to determine the distinctive characteristics for measurement of each dimension [40]. We followed a consensus process to define each dimension and identify each variable. After defining a concept based on the existing literature, we reviewed each definition and discussed to resolve any disagreement. This process was repeated until consensus was reached, and we utilized the same process to determine operational variables to complete phase six of the process.

### 3.2. Expert Panel Review Methods

For phases seven and eight, purposive sampling was used to recruit expert panel reviewers with demonstrated expertise in instrument development and/or campus food insecurity response. Recommendations for the number of expert reviewers range widely from two to more than twenty, although most recommendations fall between three and five reviewers, with goals of providing necessary expertise within the limitations of expert availabilty [41,42]. For the purpose of this study, we aimed to recruit five to ten expert reviewers to ensure expertise in relevant fields. The expert panel received copies of the draft CFAS framework to review before providing feedback through a survey administered in Qualtrics (version accessed 31 January 2021–31 January 2022; Provo, UT). Questions addressed the clarity, comprehensiveness, and representativeness of the proposed dimensions, conceptual definitions, and operational variables. The survey included multiple choice questions and written responses, with requests for expert reviewers to provide a written description of any identified issues along with recommendations for improvement. Follow-up communication with expert reviewers provided clarification when needed to ensure agreement and accuracy. Based on responses from expert panelists, items were revised to ensure clarity and comprehensiveness while maintaining the original intent of the developed framework.

## 4. Results

### 4.1. Overview of the CFAS Framework

Thematic analysis grouped sources into the following categories: academic course, advocacy, awareness efforts, centralized resources, faculty/staff involvement, financial aid, food pantry, meal scholarship, meal swipes, skills training, professional development, research and scholarship, SNAP resources, stigma, and student involvement. Sources could be included in more than one category, depending on the content of the article/source. Similar categories were combined and defined in phases three through five, resulting in the CFAS framework dimensions, definitions, and operational variables.

The CFAS framework unites Berger and Milem’s model of organizational behavior in higher education with food insecurity literature to define six dimensions of food insecurity response in higher education: Student Services and Supports; Involvement; Advocacy; Awareness and Culture Efforts; Education and Training; and Research, Scholarship, and Creative Works. The connections between Berger and Milem’s [33] model for organizational behavior in higher education and the CFAS framework for understanding college campus responses to food insecurity are described in Table 1.

#### 4.1.1. Student Services and Supports

Student Services and Supports is conceptually defined as the aid available to serve immediate and long-term food security needs, which may come in the form of food, financial, or other direct aid. This dimension encompasses the distribution of aid, including information sharing, processes for distribution, audiences for distribution, and comprehensiveness of distribution methods. Student Services and Supports addresses the bureaucratic, collegial, and symbolic dimensions of organizational behavior in higher education [33]. As bureaucracies, colleges/universities establish structures and processes to achieve internal goals, such as meeting the needs of food insecure students [33]. The collegial promotion of value among organizational members and symbolic interpretation of the quality of supports (particularly by food insecure students) demand clear communication and easy to follow processes [33].

Direct student services/supports are the most frequently implemented, researched, and recommended interventions for college student food insecurity [3,18,20,24,43]. While food pantries are most common [17], this dimension encompasses other forms of direct aid such as meal swipe sharing programs, food or dining scholarships, campus community gardens, free community meals, SNAP outreach, and emergency financial assistance.

From the perspective of food insecure college students and student support providers, the literature has identified accessibility as one of the primary barriers to accessing food assistance. This includes lack of information or unclear processes to access assistance [3,44,45], location of services [44,46], and hours of operation [17,44,45]. Furthermore, widespread communication about accessing assistance may help reduce food insecurity [8], and the presence of staff on campus has been identified as one of the primary facilitators for student SNAP enrollment [47]. Additionally, food pantry representatives have expressed concerns with maintaining quality and quantity of available items [32,46].

Based on the research described above, Student Services and Supports is operationalized to include consistency of student services/supports, hours and locations of operation, processes to access services/supports as well as efforts to inform campus members about supports, and the role of student support providers. Because unsuitable or low-quality food is a frequently identified barrier to food pantry use [44,48] and a lack of quality resources can have negative symbolic impacts on student perception [9], the ability to provide high-quality food and meet nutritional needs is also included as a facet of this dimension.

#### 4.1.2. Involvement

The Involvement dimension encompasses individuals and groups taking action to address food insecurity, the extent of their influence on campus, and coordination among actors. College campuses are diverse environments with a wide range of individuals who have skills, resources, power, experiences, or interests to contribute to food security efforts. The collegial dimension of organizational behavior emphasizes the importance of participation and cooperation among organizational members to act effectively within the more competitive political dimension [33].

The literature describes a variety of organizations, partnerships, and collaborative efforts developed in response to food insecurity [3,32,49]. Early efforts to address food insecurity on campus may be carried out by individuals acting informally to meet a need they have identified. For example, a student at Alabama Agricultural and Mechanical University started a campus food pantry out of his dorm room after noticing students going hungry [50], and faculty and administration at other campuses have created snack drawers or cabinets to help hungry students [3]. However, multiple organizations have made it possible to carry out coordinated efforts with greater impact, such as Swipe Out Hunger, College and University Food Bank Association, Campus Kitchens Project, and Food Recovery Network [16,51,52,53]. Additionally, student government associations and faculty senates, which often have campus-wide influence, have taken on efforts to support food insecure students [54,55].

The Involvement dimension is operationalized to include student and employee (faculty, staff, and administration) involvement in food security efforts. However, the specialized nature and structure of colleges/universities often leads to disciplinary silos, which can increase bureaucracy and decrease communication and collaboration [56,57]. To address the impact of silos and disintegration, Involvement also addresses coordination and collaboration efforts among individuals and groups.

#### 4.1.3. Advocacy

Advocacy is defined as “public support for an idea, plan, or way of doing something” [58]. The Advocacy dimension describes the presence of diverse campus actors who promote public support for food insecurity issues and encourage policies and practices to support food insecure students. Advocacy specifically recognizes stakeholders involved in advocacy work and the processes or policies that facilitate advocacy work.

Due to the political nature of colleges/universities, which have differing priorities and limited resources, it is essential to have dedicated campus agents acting as advocates for student needs [35]. Political pressures can be influenced by internal and external motivators from the systemic dimension of organizational behavior in higher education [33]. Also, the success of advocacy efforts is enhanced by collegial engagement with diverse individuals who can effectively utilize various forms of power and authority found on a college campus [33].

With less than one-third of college students meeting characteristics of a traditional college student [3] and the recent rise in food insecurity as a student issue, advocacy is essential to promote appropriate responses and understanding of food insecurity issues in higher education. The literature frequently mentions the importance of advocacy at campus, state, and federal levels as it relates to food insecurity [24,59]. In one article, researchers described how university administrators established a food pantry and began considering a meal scholarship program after being presented with data from a study of their campus [60]. Martinez and colleagues [61] described how university and state level advocacy led to the establishment of a food pantry on every campus in the University of California system.

The Advocacy dimension is operationalized to include the presence and reach of student and campus employee advocates. Due to the bureaucratic nature of higher education institutions, it also addresses the presence and influence of campus advocacy policies and processes. This may include open forums or other processes with the student government association or faculty senate; the presence of an ombudsman’s office; open door policies; peer advocate programs; or other similar processes or programs on campus.

#### 4.1.4. Awareness and Culture Efforts

The Awareness and Culture Efforts dimension describes actions to increase knowledge of the presence and depth of food insecurity on campus as well as efforts to confront pervasive norms and beliefs, particularly stigma, relating to food insecurity and food insecurity supports. Efforts to increase awareness of needs on campus rely on systems and relationships associated with bureaucratic and collegial dimensions in higher education [33]. According to Berger [35], clear communication is considered a component of effective bureaucratic and collegial systems, and communication plays a key role in increasing awareness of food insecurity and food insecurity supports [3,45,48].

College officials have described how a lack of awareness of student food insecurity has impeded efforts to address student need [3], and researchers have identified positive impacts from efforts to increase awareness of food insecurity on campus [62]. Efforts to increase awareness also may support normalization of food insecurity and reduce stigma [62,63]. Evidence of positive effects of awareness has resulted in frequent recommendations to increase awareness of student food insecurity among campus stakeholders [24,43,63].

Many definitions of culture exist and most commonly (and for the purpose of this framework) refer to a system or pattern of beliefs and values that guide behavior of a community or organization [64,65]. Symbolism has long been considered an explicit indication of culture [33]. Culture and symbolism are particularly relevant to stigma, which devalues individuals who carry it and is often embedded in culture [66]. Campus actions can be perceived symbolically by individuals and groups across campus based on their lens of interpretation [33]. These campus actions also influence the collegial tenets of organizational behavior in higher education by communicating value to organizational members and recognizing needs of members [33].

Stigma has been regularly associated with food insecurity. Recent studies determined that the stigma of food insecurity [43,45,67] and stigma of accessing food assistance [3,37] were major barriers to utilization of campus supports. Stigma is likely associated with many of the social and mental health impacts of food insecurity too, as students have described isolation from avoiding embarrassment or public recognition of their experience with food insecurity [9,67]. Therefore, efforts to address issues of culture associated with food insecurity and student supports are crucial to promoting utilization of resources and reducing the negative impacts of food insecurity.

The Awareness and Culture Efforts dimension is operationalized to include efforts to increase awareness of food insecurity among students and employees, which may include events, programs, and campaigns to inform campus members about the prevalence of food insecurity on campus. This dimension also addresses efforts to impact campus culture and reduce stigma of being food insecure and utilizing food insecurity services/supports. Efforts may include specialized marketing campaigns; co-location of food insecurity services with normalized services such as financial aid or academic support; or designing food insecurity supports to fit within normal campus activities.

#### 4.1.5. Education and Training

The Education and Training dimension addresses academic curriculum, professional development, and other skills-based learning opportunities to educate students and campus employees about food insecurity issues, train employees to identify and respond to student need, and equip students with skills to manage food insecurity experiences. As institutions of higher education, colleges/universities already prioritize education and training within their regular practices, making them systemic aspects of organizational behavior that are greatly influenced by their bureaucratic systems [33]. Additionally, campus employees are key to fostering connections with students, keeping students informed, and developing a sense of meaning, which are critical to the collegial dimension in higher education [33,35].

The Government Accountability Office reported educating faculty, staff, and students was a primary way colleges/universities addressed food insecurity [3]. In this dimension, student education may include traditional academic curriculum or non-curricular training opportunities. A systematic review of literature on food insecurity prevalence and response identified financial coaching as one of the most commonly recommended interventions [20]. Students also have identified education and skills training as desired supports to address food insecurity. In a study of 1093 students, food insecure students (*n* = 505) identified five supports that would be most helpful to increase access to food. Of these supports, three focused on education and training related to budget, healthy eating, and shopping for food [60]. Another qualitative study identified a desire for universities to offer financial and food literacy training to support basic needs [68]. While studies on impacts of education and skills training programs among college students are limited, one study of a college nutrition course with an integrated cooking component found a significant increase in food security and a significant decrease in very low food security among participants [69].

When considering the higher education context, it is critical to remember the role of faculty, staff, and administrators in the student experience. Many faculty and staff interact regularly with students, and they may be in an optimum position to identify at-risk students and refer them to appropriate campus resources. According to the 2019 National Survey of Student Engagement, more than half of faculty interacted with students on substantive matters outside of class [70]. In a different study, 10 percent of campus food pantry users learned about the resource through a faculty member [71]. As agents with the ability to participate in or support any actions described in this framework, professional development or training opportunities related to food insecurity can enhance the knowledge and abilities of campus employees to respond appropriately.

Because education and training can be implemented differently depending on need and campus context, the Education and Training dimension is operationalized to include multiple facets of education and training on a college or university campus. Within student education, this dimension includes undergraduate student courses and minors, graduate student courses and certificates, and student skills training opportunities. In reference to employee education, this dimension includes continuing education and professional development opportunities for faculty, staff, and administrators.

#### 4.1.6. Research, Scholarship, and Creative Works

The Research, Scholarship, and Creative Works dimension describes the conduct of research, scholarship, and/or creative works relating to food insecurity, including interdisciplinary collaboration among scholars. While the application of this dimension may differ based on bureaucratic contexts for each campus, research, scholarship, and creative works serve as uniting priorities in higher education that are core to the systemic dimension of organizational behavior in higher education [33]. In addition, interdisciplinary research, scholarship, and creative works promote the collegial relationships and dialogue necessary to bridge gaps in existing research, policy and practice [72].

Scholarly work relating to food insecurity is growing, but there are still many gaps in knowledge and understanding of food insecurity. Research/data analysis was one of the six campus initiatives identified by the GAO to address student food insecurity [3], and the literature frequently recommends scholarly work as a specific response to food insecurity, particularly interdisciplinary or integrated research [21,24]. Currently, the literature lacks substantial evaluation of existing student services/supports. Expanded research on student food insecurity prevalence and affected populations can inform programmatic, engagement, advocacy, awareness, and educational responses that make up the other five dimensions of the CFAS framework. It also can bring attention to issues of food insecurity through presentations and publications.

Like the Involvement dimension, this dimension includes collaboration to address the issue of disciplinary siloing in higher education. Given the complex nature of food insecurity, interdisciplinary research is essential to inform holistic responses to student need. The National Academy of Sciences [73], National Institutes of Health [74], and National Science Foundation [75] have prioritized interdisciplinary research with the belief it is essential for the deep understanding and innovation needed to address the world’s complex challenges.

This dimension is operationalized to address Research, Scholarship, and Creative Works by undergraduate students, graduate students, and campus employees. Activities may include traditional research, evaluation, development of evidence-based publications, creative design or writing projects, and other initiatives to add to the body of knowledge and understanding of food insecurity. These activities may be focused specifically on food insecurity among college students, or they may focus on food insecurity in additional contexts, which also has the potential to influence future responses to food insecurity among college student populations. The CFAS framework is summarized in Table 2. Operational variables are identified in Table 3 to describe campus actions associated with each dimension and develop boundaries for assessment within each dimension. Operational variables may be assessed according to evidence of practical actions taken by the campus that correspond to each variable. Examples of these actions are also included in Table 3. 

### 4.2. Expert Panel Review Results

Participating expert panelists (*n* = 7) had documented experience in instrument development and/or campus food insecurity/basic needs response. Four panelists had documented expertise in campus food insecurity response (more than 45 years of collective experience in both four-year and two-year college systems), one panelist had more than 20 years of experience in instrument development, and two panelists had experience in both subject areas (more than 30 years of collective experience). Survey responses confirmed the clarity, comprehensiveness, and representativeness of the proposed dimensions, conceptual definitions, and operational variables. A summary of expert panel responses is described in Table 4, with key changes to the framework noted. CFAS framework dimensions, conceptual definitions, and operational variables were revised to develop the final version shared in this publication.

## 5. Conclusions

Food insecurity is a known issue among college students, and many colleges/universities have taken steps to address this problem. Until now, there has been no framework to understand or assess systematic campus food insecurity response. By incorporating an existing framework for organizational behavior in higher education [33], key tenets of the socio-ecological model [27], and primary themes for campus response as identified by campus food pantry providers [32], the CFAS framework grounds itself in understanding of institutional actions driven by the priorities of campus representatives.

The CFAS framework describes six dimensions of campus food insecurity response: Student Services and Supports; Involvement; Advocacy; Awareness and Culture Efforts; Education and Training; and Research, Scholarship, and Creative Works. The dimensions comprehensively represent the many facets of college responses to food insecurity as institutions of higher education. Operational variables define campus actions within each dimension, providing metrics for assessment of campus performance. For example, if seeking to strengthen its Student Services and Supports, a campus can use the operational variables to assess the availability, accessibility, and quality of those supports. The framework provides the first evidence-based guide for understanding and assessing campus actions taken to address college student food insecurity through the lens of higher education systems. While many have sought to assess campus food pantries [44,61] or other direct supports [18,69], this framework makes it possible to assess the entirety of an institution’s response to campus food insecurity.

### 5.1. Framework Application

Designed to support assessment, researchers can use this framework to guide assessments and data collection on current campus responses. Currently, the framework is being applied as a part of Hunger Free Higher Ed (HFHE), an initiative to address college student food insecurity in Alabama. Thirty participating campuses engaged in a six-step, iterative process (Engage, Assess, Plan, Implement, Evaluate, Celebrate), with the framework being applied in the Assess and Evaluate steps [76]. While a food pantry may be the first step in response to food insecurity for many colleges/universities [17], the complexity of food insecurity calls for a multifaceted and systemic response. Therefore, this framework also can be used to inform programmatic and policy decisions at campus, state, and federal levels as campuses seek to enhance or expand their efforts to address student need. Practitioners can utilize this framework to guide discussions and development of systematic campus responses to food insecurity.

### 5.2. Limitations

Due to the lack of rigorous evaluation or national-level data associated with campus food insecurity response, this framework was developed based on available evidence, with contributions from frameworks that extend beyond food insecurity in higher education. As evidence foundations grow and best practices are identified, the framework should be revisited and adapted to align with current evidence. While the framework was developed with the intention of being used in a wide variety of campus contexts, given the wide range of campus environments and contexts, application may vary, particularly in specialized campus environments or locations outside of the United States. Finally, this framework intentionally applies only to campus responses. However, the authors recognize college student food insecurity is affected by a wide range of federal, state, and community actions that also play a key role in addressing the needs of students across the country.

### 5.3. Future Research

During the framework development process, we identified a need for greater assessment and evaluation of specific campus response efforts to inform best practices and improve processes to support food insecure college students. Further research on campus responses within the framework dimensions will help to strengthen resources, practices, and policies to ultimately reduce the prevalence and severity of food insecurity among college students.

## Figures and Tables

**Table 1:** Relationship between organizational behavior in higher education and campus food insecurity response.

Dimension of Organizational Behavior in Higher Education	Conceptual Definition	Associated Dimensions of Campus Food Insecurity Response
Bureaucratic	Emphasizes rationality in organizational decision-making through an emphasis on the use of formal structure manifested in rules, regulations, hierarchy, and goals.	- Student Services and Supports - Awareness and Culture Efforts - Education and Training - Research, Scholarship, and Creative Works
Collegial	Describes collaboration, equal participation, concern for human resources, and the use of consensus to establish goals and make other important decisions.	- Student Services and Supports - Involvement - Advocacy - Awareness and Culture Efforts - Education and Training - Research, Scholarship, and Creative Works
Political	Emerges from competition for resources and the existence of varied interests among individuals and groups within an organization.	- Involvement - Advocacy
Symbolic	Focuses on the role of symbols (e.g., stories, logos, seals, ceremonies, and traditions) in creating meaning within organizations.	- Student Services and Supports - Awareness and Culture Efforts
Systemic	Suggests that what happens inside an organization can be best understood by how the organizational system and its component sub-systems interact with and relate to broader systems in the external environment.	- Education and Training - Research, Scholarship, and Creative Works

Note. Dimensions and conceptual definitions from “Understanding the organizational nature of student persistence: Empirically-based recommendations for practice,” [35].

**Table 2:** Framework for assessing campus responses to college student food insecurity.

Dimension	Key Supporting Studies	Theoretical Definition	Conceptual Definition
Student Services and Supports	Berger & Milem, 2000 [33] Brito-Silva et al., 2022 [44] Bronfenbrenner, 1979 [27] Bruening et al., 2017 [20] El Zein et al., 2022 [48] Hagedorn-Hatfield, Richards, et al., 2022 [18] Olfert et al., 2021 [8] Reppond et al., 2018 [32]	Direct student services/supports are the most frequently implemented, researched, and recommended interventions. Efforts to increase accessibility of resources have been identified as important factors to ensure utilization of services/supports by students in need.	Describes aid available to serve immediate and long-term food security needs, which may come in the form of food, financial, or other direct aid. The distribution of aid includes information sharing, processes for distribution, audiences to whom it is distributed, and the comprehensiveness of distribution methods.
Involvement	Berger & Milem, 2000 [33] Bronfenbrenner, 1979 [27] GAO, 2018 [3] Phillips et al., 2018 [49] Savoca & Bishop, 2020 [56]	College/university campuses are diverse and complex environments with a variety of actors with potential to contribute to food security efforts. Increased involvement from diverse campus actors can aid greatly in efforts to minimize food insecurity on campus.	Describes individuals and/or groups taking action to address food insecurity, the extent of their influence, and coordination among actors.
Advocacy	Berger, 2001 [35] Berger & Milem, 2000 [33] Bronfenbrenner, 1979 [27] Landry et al., 2022 [59] Thompson et al., 2019 [24]	Due to the political nature of campuses and varying perceptions of college student needs, research has indicated the importance of having students and employees at multiple levels and positions on campus who are aware of campus food security issues and advocating on behalf of student needs.	Describes the presence of diverse campus actors who promote awareness of food insecurity issues and encourage policies and practices to support food insecure students.
Awareness and Culture Efforts	Berger, 2001 [35] Berger & Milem, 2000 [33] Bronfenbrenner, 1979 [27] El Zein et al., 2018 [45] Hagedorn Hatfield, Hood, et al., 2022 [63] Olfert et al., 2020 [62] Weaver et al., 2022 [37]	Increased campus awareness and understanding of food insecurity are critical to improving and establishing programs for food insecure and at-risk students. It also can influence stigma, which has been described as a barrier to accessing food security supports as well as a potential contributor to mental health impacts of food insecurity.	Describes efforts to increase knowledge of the presence and depth of food insecurity on campus as well as efforts to confront pervasive norms and beliefs, particularly stigma, relating to food insecurity and food insecurity supports.
Education and Training	Berger & Milem, 2000 [33] Bronfenbrenner, 1979 [27] Esaryk et al., 2021 [71] GAO, 2018 [3] McArthur et al., 2018 [60] National Survey of Student Engagement, 2020 [70]	Education and training are core tenets of higher education and common elements of food insecurity response. Education and training related to food insecurity are vital to increasing understanding of food insecurity issues and equipping stakeholders to respond appropriately to student need based on their position or situation.	Describes academic curriculum, professional development, and other skills-based learning opportunities to educate students and campus employees about food insecurity issues, identifying and responding to student need, and skills to manage food insecurity experiences.
Research, Scholarship, and Creative Works	Berger & Milem, 2000 [33] Bronfenbrenner, 1979 [27] GAO, 2018 [3] Holben & Marshall, 2017 [21] Thompson et al., 2019 [24]	Research, scholarship, and/or creative works are primary functions of higher education institutions. By examining food insecurity issues, colleges/universities can inform the resources, practices, and policies that address food insecurity on campus and bring attention to the issue through presentations and publications.	Describes the conduct of research, scholarship, and/or creative works relating to food insecurity, including interdisciplinary collaboration among researchers/scholars.

**Table 3:** Operational variables for assessing campus responses to college student food insecurity.

Dimension	Operational Variables	Example of Variable Application
Student Services and Supports	- Consistency of emergency student services/supports - Consistency of long-term student services/supports - Hours of operation for student services/supports - Locations of student services/supports - Processes to access student services/supports - Efforts to inform students of available services/supports - Efforts to inform faculty, staff, and administration of available student services/supports - Ability to meet nutrition needs of food insecure students - Student services/support providers	- Variable: Efforts to inform students of available services/supports - Application: Extent, frequency, and reach of student-facing communication about food insecurity services (e.g., campus-wide messaging, repeated outreach, web presence)
Involvement	- Student involvement - Faculty, staff, and administrator involvement - Coordination of involved actors	- Variable: Student involvement - Application: Level and scope of student engagement in food insecurity efforts
Advocacy	- Presence and reach of student advocates - Presence and reach of faculty, staff, and administrator advocates - Student advocacy processes and policies - Faculty, staff, and administrator advocacy processes and policies	- Variable: Student advocacy processes and policies - Application: Existence and utilization of processes and/or policies that enable student advocacy associated with food security needs
Awareness and Culture Efforts	- Efforts to increase student awareness of food insecurity - Efforts to increase faculty, staff, and administrator awareness of food insecurity - Efforts to reduce food insecurity stigma - Efforts to reduce the stigma of utilizing food insecurity services/supports	- Variable: Efforts to reduce food insecurity stigma - Application: Presence, frequency, and reach of initiatives to reduce stigma associated with food insecurity
Education and Training	- Undergraduate student courses - Undergraduate student minor - Graduate student courses - Graduate student certificate - Student skills training opportunities - Continuing education and professional development opportunities for faculty, staff, and administrators	- Variable: Student skills training opportunities - Application: Existence and reach of student-focused skills training associated with food insecurity (e.g., financial literacy, food preparation)
Research, Scholarship, and Creative Works	- Undergraduate student engagement in research, scholarship, and creative works - Graduate student engagement in research, scholarship, and creative works - Faculty, staff, and administrator involvement in research, scholarship, and creative works	- Variable: Graduate student engagement in research, scholarship, and creative works - Application: Existence and extent of graduate student involvement in food security-related research, scholarship, and/or creative works

Note: Examples illustrate potential indicators for applied assessment and are not intended as exhaustive or prescriptive measures.

**Table 4:** Summary of expert panel reviewer responses.

Question	Expert Panel Response (*n*)	Notes and Key Adjustments
Is theoretical basis appropriate and comprehensive?	Yes (6) No (1)	The panelist noted faculty should be included in multiple dimensions due to their regular interface with students. In the final version, faculty are included in operational variables for Involvement; Advocacy; Awareness and Culture Efforts; Education and Training; and Research, Scholarship, and Creative Works.
Is conceptual definition clear and reflective of theoretical basis?	Yes (7) No (0)	--
Is each dimension representative of content domain?	Yes (7) No (0)	--
Is each dimension exhaustive of theoretical framework?	Yes (6) No (1)	The panelist recommended personal and racial bias training be included in the Education and Training dimension. While the final version does not explicitly mention personal and racial bias, the conceptual definition was expanded to be inclusive of training that addressed issues related to effective and comprehensive food insecurity response.
Is each dimension mutually exclusive within theoretical framework?	Yes (4) No (2) No response (1)	Both panelists who answered No recognized Campus Culture and Awareness Efforts may have a relationship to actions in other dimensions. However, neither panelist described this as a weakness. Both panelists encouraged leaving it as a standalone dimension, with the recommendation to consider parent concepts in the analysis of future framework applications.

## Data Availability

The original contributions presented in this study are included in the article. Further inquiries can be directed to the corresponding author(s).
